# Supplementary material for: Pathomorphological Findings and Infectious Diseases in Selected European Brown Hare (Lepus europaeus Pallas, 1778) Populations from Schleswig-Holstein, Germany
Source: Pathogens. 2023 Nov 5;12(11):1317. doi: 10.3390/pathogens12111317 (PMC10675426; doi:10.3390/pathogens12111317)
Supplement: Supplementary file 1 [file pathogens-12-01317-s001.zip › pathogens-2648995-supplementary.pdf]

Table S1. Assessment of significance of terms in the highest-ranking model for each pathology. The estimate is the coefficient on the linear predictor scale. Exponentiating the estimate gives the odds ratio. Df denotes degrees of freedom. Enteritis was not observed in 2019, so that the effect of year could not be quantified for 2019. The asterisks "*" mark significant *p*-values.

Term	Estimate	Std. Error	Odds ratio	Df	Deviance	*p*-value
**Model: hepatitis ~ age + year**						
age (reference level: juvenile)				1	−9.53	0.002*
adult	1.2	0.4	3.31			
Year (reference level: 2016)				3	−19.61	0.0002*
2017	−1.91	0.47	0.15			
2019	−1.23	0.53	0.29			
2020	−1.42	0.54	0.24			
**Model: enteritis ~ origin + year**						
Origin (reference level: Elpersbuettel)				2	−4.65	0.097
Friedrichskoog	−0.65	0.69	0.52			
Tetenbuell	0.63	0.57	1.88			
year (reference level: 2016)				3	−9.2	0.027*
2017	0.23	0.54	1.26			
2019	not quantifiable					
2020	−0.055	0.68	0.95			
**Model: parasites_in_intestine ~ age*sex**						
age (reference level: juvenile)						
adult	0	0.5	1			
sex (reference level: female)						
male	0.79	0.64	2.2			
age:sex				1	−3.9	0.048*
adult:male	−1.48	0.76	0.23			
**Model: pneumonia ~ sex**						
sex (reference level: female)				1	−5.23	0.022*
male	−2.01	1.08	0.13			
**Model: nephritis ~ age + year**						
age (reference level: juvenile)				1	−6.62	0.01*
adult	1.08	0.44	2.93			
year (reference level: 2016)				3	−12.25	0.0066*
2017	−1.52	0.48	0.22			
2019	−1.15	0.58	0.32			
2020	−0.75	0.51	0.47			

Table S2. Posthoc comparisons for significant predictors in the logistic regression models. The asterisks "*" mark significant *p*-values.

Comparison	Estimate	Std. Error	Odds Ratio	z value	Adjusted *p*-value
**For variable year in model hepatitis ~ age + year**					
2017 vs 2016	−1.91	0.47	0.15	−4.03	0.00033*
2019 vs 2016	−1.23	0.53	0.29	−2.32	0.082
2020 vs 2016	−1.41	0.54	0.24	−2.64	0.041*
2019 vs 2017	0.68	0.53	1.97	1.28	0.61
2020 vs 2017	0.49	0.5	1.63	0.99	0.65
2020 vs 2019	−0.19	0.59	0.83	−0.32	0.75
**For variable year in model enteritis ~ origin + year**					
2017 vs 2016	0.23	0.54	1.26	0.43	1
2020 vs 2016	−0.055	0.68	0.95	−0.081	1
2020 vs 2017	−0.29	0.67	0.75	−0.43	1
**For variables sex and age in model parasites in intestine ~ age*sex**					
male adult vs male juvenile	−1.48	0.57	0.23	−2.6	0.038*
female adult vs female juvenile	0	0.5	1.00	0	1
male adult vs female adult	−0.69	0.41	0.50	−1.69	0.27
male juvenile vs female juvenile	0.79	0.64	2.20	1.23	0.44
**For variable year in model nephritis ~ age + year**					
2017 vs 2016	−1.52	0.48	0.22	−3.19	0.0086*
2019 vs 2016	−1.15	0.58	0.32	−1.97	0.25
2020 vs 2016	−0.75	0.51	0.47	−1.47	0.56
2019 vs 2017	0.37	0.65	1.45	0.58	1
2020 vs 2017	0.77	0.56	2.16	1.38	0.56
2020 vs 2019	0.4	0.67	1.49	0.59	1

Table S3. Pathomorphological findings of European brown hares (*n* = 155) and their yearly distribution. Total percentages are given only for routinely collected histopathological samples or macroscopic detectable alterations. Chosen relevant pathomorphological findings are outlined in bold.

**Pathomorphological Findings**		**2016**	**2017**	**2019**	**2020**	**Total**	**Total %**
**Alimentary system**							
Liver and biliary tract	Bile duct proliferation	6	1	1		8	5.2
	Cholangitis		1			1	0.6
	Dystrophic calcification		1	1		2	1.3
	**Hepatitis**	**41**	**19**	**12**	**14**	**86**	**55.5**
	Liver cell necrosis	7	2	1	3	13	8.4
	Liver fibrosis	9	3	2		14	9.0
Intestine	**Enteritis**	**8**	**9**		**4**	**21**	**13.5**
	Unformed rectal content		19	19	12	50	32.3
	**Parasites in intestine**	**35**	**33**	**14**	**17**	**99**	**63.9**
	Serositis	1				1	0.6
Stomach	Gastritis				2	2	1.3
**Cardiovascular system**							
Heart	Endocardial haemosiderosis	1				1	0.6
	Myocarditis	1				1	0.6
**Abdominal and thoracic cavity**							
Peritoneum	Dystrophic calcification			1		1	0.6
	Fat tissue mineralisation	2	1			3	1.9
	Parasitic cyst		1			1	0.6
	**Steatitis**	**11**	**15**	**4**	**6**	**36**	**23.2**
Pleura	Dystrophic calcification			1		1	0.6
	Fibrosis	1				1	0.6
	Pleuritis	1	2			3	1.9
**Endocrine and haematopoietic system**							
Adrenal glands	Adrenalitis	1	1			2	1.4
	Cyst	1				1	0.7
	Hyperplasia		2			2	1.4
Lymph nodes	Lymphadenitis of mesenteric lymph nodes	1	1			2	1.3
	Mesenteric lymph node/ Peyer´s patches hyperplasia	17	19	11	14	61	39.4
	Mesenteric lymph node/ Peyer´s patches mineralisation	4	8		5	17	11.0
	Pulmonary lymph node hyperplasia			4	2	6	
Spleen	Hyperplasia	20	18	2	3	43	27.7
	Mineralisation	2				2	1.3
**Nervous system**							
Cerebral ventricles	Plexus choroiditis	1				1	0.7
**Respiratory system**							
Lung	**Pneumonia**	**2**	**2**	**3**	**1**	**8**	**5.2**
Trachea	Tracheitis				2	2	
**Skin**							
Skin	Dermatitis				1	1	
**Urinary and genital system**							
Kidneys	**Nephritis**	**25**	**9**	**5**	**9**	**48**	**31.0**
	Fibrosis				2	2	1.3
Testes	Orchitis		1			1	
Ovary	Oophoritis		1			1	

Uterus	Perimetritis	1	1

Table S4. Detected bacterial and fungal microorganisms with regard to their organ localisation in deceased hares.

**Bacteria/ Fungi**	**Intestine**	**Kidney**	**Liver**	**Lung/Trachea**	**Reproductive tract**	**Spleen**	**Mesenteric lymph node**	**Total**
Acinetobacter spp.	4			6				10
Aeromonas encheleia			1	1				2
Aeromonas eucrenophila				1				1
Aeromonas spp.	11	1	6	50	2			70
Bacillus spp.	3	2	3	28	2			38
Bacteroides cellulosilyticus	1						2	3
Bacteroides ovatus	1			1				2
Bacteroides thetaiotaomicron				1				1
Bacteroides uniformis							1	1
Bacteroides vulgatus	2			1				3
Bordetella bronchiseptica				1				1
Brucella suis Biovar 2			2					2
Buttiauxella spp.				6				6
Candida albicans	1							1
Candida famata	2			1				3
Carnobacterium maltaromaticum				1				1
Chryseobacterium indologenes			2	1				3
Chryseobacterium joostei			1		1			2
Chryseobacterium spp.				3				3
Citrobacter amalonaticus				1				1
Citrobacter gillenii				3				3
Coliform bacteria				4				4
Coryneform bacteria (not determinable)				4				4
Enterobacter cloacae		1	1	12				14
Enterobacter spp.	3	1		22				26
Enterococcus faecalis	1			2				3
Enterococcus spp.	1		1	32	1			35
Erwinia rhapontici				1				1
Erwinia spp.	2			3				5
Escherichia coli	128	2	8	118	2	2	6	266
Escherichia coli var. *haemolytica*	6			4				10
Ewingella americana	1			7				8
Fungi (not determinable)	1		1					2
Hafnia alvei			1					1
Klebsiella oxytoca	1			1				2
Klebsiella spp.				3				3
Kluyvera intermedia				2				2
Kluyvera spp.	1							1
Kocuria spp.							1	1
Kosakonia cowanii				2				2
Lactococcus spp.				3				3
Leclercia adecarboxylata				2				2

**Bacteria/ Fungi**	**Intestine**	**Kidney**	**Liver**	**Lung/Trachea**	**Reproductive tract**	**Spleen**	**Mesenteric lymph node**	**Total**
Leclercia spp.					1			1
Lelliottia amnigena	1			7				8
Listeria ivanovii	1							1
Myroides spp.				1				1
Non-fermenting, gram-negative bacteria (not determinable)	1			11				12
Pantoea agglomerans				7				7
Pantoea spp.				6				6
Pseudomonas brenneri				1				1
Pseudomonas fragi				7				7
Pseudomonas koreensis				4				4
Pseudomonas libanensis				1				1
Pseudomonas lundensis			1	9				10
Pseudomonas putida	1			6				7
Pseudomonas spp.	19	1		77	2	1		100
Pseudomonas taetrolens				2				2
Pseudomonas synxantha				1				1
Rahnella aquatilis	6	1	1	6				14
Raoultella ornithinolytica				1				1
Raoultella terrigena				1				1
Serratia fonticola	1			9				10
Serratia liquefaciens	2			6				8
Serratia marcescens				1				1
Serratia plymuthica				1				1
Serratia proteamaculans				1				1
Serratia spp.	1			11				12
Shewanella baltica				2				2
Shewanella spp.			1	2				3
Sphingobacterium spp.				3				3
Staphylococcus equorum		1						1
Staphylococcus spp.				1				1
Staphylococcus spp., coagulase-negative				6		1		7
Stenotrophomonas maltophilia				2				2
Stenotrophomonas spp.				1				1
Streptococcus spp., α-haem.		1	5	44	2			52
Streptococcus spp., γ-haem.	2			17				19
Streptococcus ferus				3				3
Yarrowia lipolytica	3			3				6
Yeast (not further determined)	21		4	13		1		39
Yersinia enterocolitica	1							1
**Total**	**230**	**11**	**39**	**602**	**13**	**5**	**10**	**910**
